# Layered Double Hydroxide Nanomaterials Encapsulating *Angelica gigas* Nakai Extract for Potential Anticancer Nanomedicine

**DOI:** 10.3389/fphar.2018.00723

**Published:** 2018-07-09

**Authors:** Hyoung-Jun Kim, Gyeong Jin Lee, Ae-Jin Choi, Tae-Hyun Kim, Tae-il Kim, Jae-Min Oh

**Affiliations:** ^1^Department of Chemistry and Medical Chemistry, College of Science and Technology, Yonsei University, Wonju, South Korea; ^2^Department of Biosystems & Biomaterials Science and Engineering, College of Agriculture and Life Sciences, Seoul National University, Seoul, South Korea; ^3^Department of Agrofood Resources, National Institute of Agricultural Sciences of RDA, Wanju, South Korea; ^4^Department of Physics, Chemistry and Pharmacy, University of Southern Denmark, Odense, Denmark; ^5^Research Institute of Agriculture and Life Sciences, Seoul National University, Seoul, South Korea

**Keywords:** layered double hydroxide, *Angelica gigas* Nakai, anticancer activity, decursin species, nanobiohybrid, phytochemical

## Abstract

We prepared hybrids consisting of *Angelica gigas* Nakai (AGN) root or flower extract and layered double hydroxide (LDH) for potential anticancer nanomedicine, as decursin species (DS) in AGN are known to have anticancer activity. Dimethylsulfoxide solvent was determined hybridization reaction media, as it has affinity to both AGN and LDH moiety. In order to develop inter-particle spaces in LDH, a reversible dehydration-rehydration, so-called reconstruction route, was applied in AGN-LDH hybridization. Quantitative analyses on AGN-LDH hybrids indicated that the content of DS was two times more concentrated in the hybrids than in extract itself. Using X-ray diffraction, FT-IR spectroscopy, scanning electron microscopy, and zeta-potential measurement, we found that AGN extract moiety was incorporated into inter-particle spaces of LDH nanoparticles during the reconstruction reaction. Time-dependent DS release from hybrids at pH 7.4 (physiological condition) and pH 4.5 (lysosomal condition) exhibited a pH-dependent release of extract-incorporated LDH hybrids. An anticancer activity test using HeLa, A549, and HEK293T cells showed that the AGN-LDH hybrid, regardless of extract type, showed enhanced anticancer activity compared with extract alone at an equivalent amount of DS, suggesting a nanomedicine effect of AGN-LDH hybrids.

## Introduction

Herbal medicine containing diverse bio-active phytochemicals have long been utilized (Kesarwani and Gupta, 2013; Baumann, 2014). Recently, medicinal researchers have tried to find out therapeutic single component (Lim et al., 2001); however, several studies suggested that whole extract rather than single component is advantageous in therapies as complicated components stimulate multiple targets, induce synergetic effect and act as bio-enhancers (Hu et al., 2009). Therefore, improved drug efficacy could be envisaged with the less-purified extract (Lee et al., 2009; Rasoanaivo et al., 2011).

*Angelica gigas* Nakai (AGN), one of the herbal crops widely distributed in eastern Asia, has long been utilized as a herbal medicine source. Its root extract in particular has been utilized to treat anemia, hypertension, arthritis, menstrual disorders, etc. (Suzuki et al., 2002; Song et al., 2004; Circosta et al., 2006; Guo et al., 2007; Kil et al., 2008). According to recent researches, roots, stems, and leaves of AGN contain bio-active substances such as pyranocoumarins (decursin, decursinol angelate, prantschimgin, angelinol, agasyllin, etc.), flavonoids, polysaccharides, phenolics, and volatile aromatics (Zhang et al., 2012). We verified in our previous study that the flowers of AGN, which have not been utilized previously as a herbal medicine resource, are also rich in antioxidants, such as phenolic acids and flavonoids (Kim et al., 2015). Among those phytochemicals, the decursin species (DS: decursin and decursinol angelate) have been shown to have excellent anticancer activity in prostate cancer (Jiang et al., 2006; Guo et al., 2007). Similar to other herbal medicine-based studies, DS administered with other extract components showed higher anticancer efficacy on lung and prostate cancers compared with DS only (Lee et al., 2009).

In this sense, a natural extract containing bio-active substances and bio-enhancers is expected to show improved drug efficacy over purified single molecules. However, there are still problems with herbal medicine in that the content of active substances responsible for the major functions is very low. According to our previous study, a methanol extract of AGN root contains only 8.5 wt/wt% of antioxidant (phenolic acid and flavonone) and 0.2 wt/wt% of anticancer DS species (Kim et al., 2016). Thus, a high dose of AGN extract would be required for practical application.

In order to overcome these drawbacks, strategies of nanomedicine could be taken. Nanomaterials such as liposomes, polymeric particles, dendrimers, and inorganic materials have been utilized as nanomedicine vehicles. They can encapsulate drugs using their appropriate internal spaces and adsorb drugs on large surface to protect vulnerable molecules from external harsh condition (Oh et al., 2006b). They can rearrange drug molecules in their interior to modify solubility (Mohammed et al., 2004), to improve bioavailability and to release drug in controlled manner (Maincent et al., 1986; Kang et al., 2015). Especially, pH responsive drug release is possible in several vehicles utilizing nanomaterials’ pH-dependent properties (Kim et al., 2007). Several nanomaterials were developed to deliver drug molecules to target organs using surface modified nanomaterials as targeting ligands (Oh et al., 2009). In this way, nanomedicine can reduce the administration dose of drug molecules. Among various nanomaterials, layered double hydroxides (LDHs), which can characteristically encapsulate bio-active molecules in their 2-dimensional interlayer space, are potential drug delivery carriers. LDHs have a layer-by-layer stacked structure consisting of a positive layer (M^2+^_1−x_M^3+^_x_(OH)_2_^x+^: ∼0.5 nm thickness, hundreds of nanometers in diameter) and exchangeable anions (A^n−^_x/n_) (Rives, 2001). Interestingly, the interlayer space of LDHs can be as small as 0.37 nm with an interlayer of NO_3_^−^ and be freely expanded to a few nanometers with bulky organic species (Cavani et al., 1991; Rives, 2001). Thus, LDHs have been reported to stabilize fragile biomolecules (Oh et al., 2006b), to solubilize poorly water-soluble drugs (Berber et al., 2008), to release incorporated drug moieties in a controlled manner (Kang et al., 2015), and to transport massive amounts of drug molecules into intracellular systems (Oh et al., 2006c). LDHs have several more characteristic advantages as delivery carriers. LDHs are mechanically more stable than organic-based carriers like liposomes. They are less cytotoxic and more biocompatible than the other nanoparticles such as iron oxides and carbon nanotubes (Choi et al., 2009). They can be dissolved into simple ions at weakly acidic condition (possibly lysosomal condition), while organic carriers produce various sized residues upon hydrolysis. In terms of natural extract incorporation, LDHs can be effective in that (i) their interlayer nanospace and large inter-particle space provide appropriate spaces for variously sized natural extract molecules (Rives, 2001; Kang et al., 2015), and (ii) positive layer charges have a high affinity for phytochemicals, which usually have a negative charge or δ^−^ sites (Rives, 2001; Kim et al., 2016).

In this study, we encapsulated whole natural extract from the root or flower of AGN into LDH nanomaterials. We tried to incorporate most of the phytochemical substances of the natural extracts into LDH nanomaterials, considering the bio-enhancer function. In our previous study, we showed the possibility to load extract moiety into LDH hybrids (Kim et al., 2016). However, that hybrid contained quite low amount of active components (DS), and thus it is strongly required to enhance DS content for practical applications as anticancer nanomedicine. In this regard, we designed encapsulation conditions to optimally concentrate DS in LDH nanomedicine. Solvent conditions were carefully selected in order to solubilize both hydrophilic and hydrophobic natural extract molecules and to facilitate the incorporation of the organic moieties such as carbohydrates, lipids, flavonoids, and DS in AGN extract into LDH nanomaterials. The encapsulation method was strategically chosen to maximize incorporation efficiency and to enhance the affinity for cellular membranes. LDHs can undergo a reversible dehydration-rehydration process, called reconstruction (Hibino and Tsunashima, 1998). Thermal treatment of LDH nanomaterials at ∼400°C produces a mixed metal oxide that can recover the original LDH structure in the presence of water and anionic species to be encapsulated (Nyambo et al., 2009). This encapsulation strategy forms particle nanostructures that are less crystalline and highly interconnected, resulting in inter-particle space, which can accommodate various organic substances (Kang et al., 2015), while the external surface of an organic-LDH hybrid possesses a positive charge for effective interactions with negative cellular membrane (Oh et al., 2006a). The goal of this study is to investigate the structure of the obtained hybrid nanomedicine and to evaluate their potential for anticancer treatment. In this regard, DS concentration effect and controlled release by LDH nanomaterials were investigated and the anticancer properties of the hybrid nanomedicines were evaluated in several cancer cells.

## Materials and Methods

### Materials

Root and flower extracts of *Angelica gigas* Nakai (AGN) were provided by the National Institute of Horticultural and Herbal Science in Korea. Magnesium nitrate hexahydrate [Mg(NO_3_)_2_⋅ 6H_2_O], aluminum nitrate nonahydrate [Al(NO_3_)_3_⋅9H_2_O], sodium bicarbonate (NaHCO_3_), and dimethylsulfoxide (DMSO) were purchased from Sigma-Aldrich Co. LLC. (St. Louis, MO, United States). A standard decursin sample for chromatography was purchased from ChromaDex Inc. (Irvine, CA, United States). Sodium hydroxide (NaOH), n-hexane, and chloroform were acquired from DAEJUNG CHEMICALS & METALS CO. LTD. (Siheung, South Korea). Methanol (Burdick & Jackson^®^, Morristown, NJ, United States) and acetone (SAMCHUN CHEMICAL CO., Yeosu, South Korea) were used to solubilize AGN extract solid. Hanks’ balanced salt solution (HBSS) was obtained from WELGENE Inc. (Taipei, Taiwan). Phosphate buffered saline (PBS) was acquired from Lonza (Walkersville, MD, United States). All reagents were used without further purification.

### Solubility of DS in Various Solvents

Dried AGN extract (200 mg), which was previously extracted in methanol at 25°C and dried by vacuum evaporation at 60°C, was dissolved in six different solvents (100 mL) including deionized water (DW), methanol, DMSO, acetone, n-hexane, and chloroform. The DS content in each solution was quantified by high performance liquid chromatography (HPLC, Yonglin YL9100 Series, Anyang, South Korea). The mobile phase was an acetonitrile:DW (6:4) mixture with a flow rate of 0.8 mL/min, and a UV-Vis detector with a wavelength of 350 nm was used.

### Preparation of Pristine LDH (MgAl-LDH) and Its Calcined Form

The pristine MgAl-LDH was prepared by a conventional coprecipitation method followed by hydrothermal treatment, as reported elsewhere (Kim et al., 2011). Mg(NO_3_)_2_⋅6H_2_O (0.06 mol) and Al(NO_3_)_3_⋅9H_2_O (0.03 mol) were dissolved in 200 mL of DW, and the solution was titrated to pH ∼9.5 with NaOH/NaHCO_3_ (0.23 mol/0.17 mol in 250 mL DW). Then, the white suspension was transferred to a Teflon-lined autoclave and put in an oven at 150°C for 24 h. Finally, a white precipitate (MgAl-LDH) was collected by centrifugation, thoroughly washed with DW, and then lyophilized. In order to prepare the calcined LDH, the MgAl-LDH powder was put in an alumina boat and thermally treated at 400°C in an electric muffle furnace for 9 h.

### Preparation of AGN-LDH Hybrid by Reconstruction

AGN extracts, both from root (RT) and flower (FL) (27.1 mg and 36.6 mg, respectively), were dissolved in 1 mL of DMSO, and then 30 mL of calcined LDH in water suspension (23 mg/mL) was added to the AGN/DMSO solutions. After thorough mixing, 70 and 44 mL of DW was added to the mixtures for RT and FL, respectively, to reconstruct the LDH structure. The reactants were stirred for 12 h in darkness. Then, the suspension was centrifuged, washed with DW, and lyophilized. The obtained hybrids were designated as RT-LDH and FL-LDH for AGN root extract-incorporated hybrid and flower extract-incorporated hybrid, respectively.

### Characterization

The crystal structures of MgAl-LDH, its calcined form, and the hybrids were investigated by powder X-ray diffraction (PXRD, D2 Phaser with LYNXEYE^TM^ detector, Bruker AXS GmbH, Kalsruhe, Germany) with Cu K_α_ radiation (λ = 1.5406 Å). The ground powder samples were mounted on a poly(methyl methacrylate) holder, and a diffraction pattern was obtained in the 2𝜃 range of 3–80° with scanning steps of 0.02°. The chemical property of MgAl-LDH, AGN extract and the hybrids was confirmed by Fourier transform-infrared spectroscopy (FT-IR, spectrum one B.v5.0 spectrometer, Perkin Elmer, Waltham, MA, United States) utilizing conventional KBr method. The morphology and particle size of MgAl-LDH, its calcined form, and the hybrids were observed with field emission-scanning electron microscopy (FE-SEM, Quanta 250 FEG, FEI, Hillsboro, OR, United States). For the SEM measurement, the powder samples were immobilized on carbon tapes and sputtered with Pt/Pd for 30 s. For the zeta-potential measurement, 1 mg of MgAl-LDH and each hybrid powder were dispersed in 1 mL DW, and then the zeta-potential was measured by ELSZ-1000 (Otsuka, Kyoto, Japan). The contents of AGN (RT or FL) in corresponding hybrids were evaluated by measuring the weight difference of the organic moiety before and after the hybridization reaction. DS was quantified with HPLC as described above.

### DS Release Study

In order to evaluate the amount of DS released from the hybrids, 50 mg of hybrid powder was dispersed into 100 mL of media such as pH 7.4 PBS or pH 4.5 HBSS. At the determined time point (5, 10, 15, 20, 25, 30, 60, 90, or 120 min), 1 mL of suspension was collected, and the solid part was removed by centrifugation at 8000 rpm and filtration with a 0.45-μm pore syringe filter (Advantec Toyo Roshi Kaisha Ltd., Tokyo, Japan). The fractional release of DS was quantified with an HPLC, as described above. All experiments were performed in triplicate. The observed time-dependent release was fitted with four kinds of kinetic models including the first-order kinetic (Eq. 1), Elovich model (Eq. 2), parabolic diffusion (Eq. 3), and power function (Eq. 4), as shown below (Jalali, 2006; Shariatmadari et al., 2006).

(1) $$\text{First order ln}Q_{\text{t}}=\text{ln}Q_{\text{e}}-k_{1}t$$

k_1_: first-order rate constant (h^−1^)

(2) $$\text{Elovich equation }Q_{\text{t}}=a+b\ln t$$

*a*: released quantity in the initial phase, *b*: release rate

(3) $$\text{Parabolic diffusion }Q_{\text{t}}=Q_{\text{e}}+R{\text{t}}^{1/2}$$

*R*: diffusion rate constant

(4) $$\text{Power function }Q_{\text{t}}={\text{αt}}^{\text{β}}$$

α: initial desorption rate constant, β: desorption rate coefficient

*Q*_t_ is the released amount after *t* min, and *Q*_e_ is the released amount at equilibrium for all models.

### Cell Culture

Human cervical adenocarcinoma cells (HeLa), human lung adenocarcinoma epithelial cells (A549), human embryonic kidney cells containing SV40 Large T-antigen (HEK293T), and mouse myoblast cells (C2C12) were grown in DMEM supplemented with 10% FBS and 1% penicillin/streptomycin in a humidified atmosphere containing 5% CO_2_ at 37°C.

### Trypan Blue Assay of AGN Extracts and AGN-LDH Hybrids

Anticancer activity of AGN extract and AGN-LDH hybrids was evaluated by trypan blue assay. Conventional MTT assay was not used because DS can disturb the absorbance of formazan in MTT assay. Cells were seeded at a density of 2.0 × 10^5^ cells per well on 6-well culture plates. After 24 h of incubation, sample solutions or suspensions (0.1% DMSO/serum-containing DMEM) were added to the cells at pre-determined DS concentrations for 24 h. Then, cells were trypsinized and collected in PBS. After centrifugation (100 g, 5 min), cell pellets were resuspended in 1 mL PBS and stained with 0.4% trypan blue solution for 2 min at room temperature (Strober, 2001). The numbers of stained dead cells and unstained viable cells among 200–300 cells were counted with a hemacytometer each time. All experiments were performed in triplicate.

## Results

### Solubility of DS in Various Solvents

**FIGURE 1:**
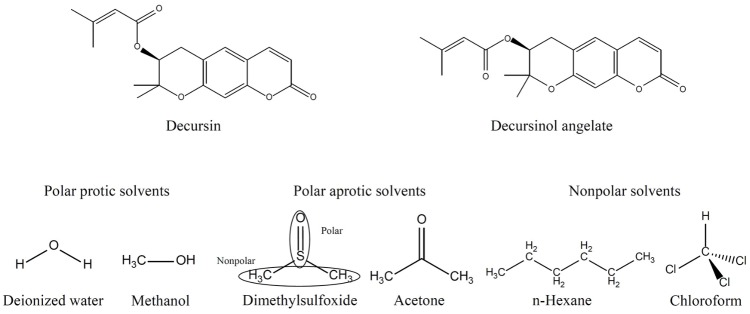
Molecular structures of DS and secondary extraction solvents. DS, decursin species.

**Table 1** displays the solubility of extracts and DS (**Figure 1**) in various solvents. Dried AGN methanol extract was dissolved in several solvents: polar protic solvents (water, methanol), polar aprotic solvents (DMSO, acetone), and non-polar solvents (n-hexane, chloroform) (**Figure 1**). In terms of dissolution efficacy for total extract components, methanol, DMSO, and deionized water showed excellent capacities. However, water did not effectively solubilize DS. Considering both total extract and DS dissolution, DMSO showed the best ability and thus was selected as the hybridization reaction media.

**Table 1:** Dissolved extract and DS from 200 mg of *Angelica gigas* Nakai root methanol extract.

Solvent type	Extraction solvent	Dissolved extract (mg)	Dissolved DS (mg)
Polar protic	Deionized water	137 ± 39	2.5 ± 0.01
	Methanol	202 ± 24	40.3 ± 0.54
Polar aprotic	Acetone	76 ± 8.9	34.1 ± 0.59
	Dimethylsulfoxide	192 ± 25	58.4 ± 7.48
Non-polar	n-Hexane	57 ± 11	31.5 ± 0.62
	Chloroform	61 ± 17	27.2 ± 0.68

*DS, decursin species.*

### Characterization of AGN-LDH Hybrids

**FIGURE 2:**
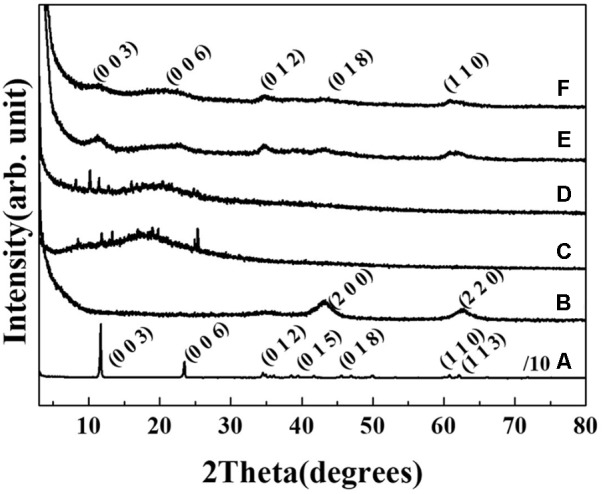
Powder X-ray diffraction patterns of **(A)** MgAl-LDH, **(B)** calcined LDH, **(C)** root extract powder, **(D)** flower extract powder, **(E)** RT-LDH hybrid, and **(F)** FL-LDH hybrid. LDH, layered double hydroxide; RT, root; FL, flower.

The crystal structures of the two AGN-LDH hybrids, RT-LDH and FL-LDH, were analyzed and compared with those of pristine LDH and the extract itself by measuring powder X-ray diffraction patterns (**Figure 2**). Pristine LDH showed sharp diffraction peaks at 2𝜃 values of 11.7, 23.5, 34.6, 38.5, 45.5, 60.8, and 62.2°, which could be assigned to (hkl) indexes of (003), (006), (012), (015), (018), (110), and (113), respectively, showing the intended hydrotalcite (JCPDS (Joint Committee on Powder Diffraction Standards) card No. 14-0191) phase. Calcined LDH exhibited (200) and (220) peaks at 2𝜃 43.3 and 62.5°, suggesting the evolution of MgO (periclase, JCPDS card No. 45-0946) upon thermal treatment. In contrast, two kinds of extracts from RT and FL showed an amorphous phase with some salt-like peaks in XRD patterns. After hybridization, the crystal structure of LDH was observed to be recovered, showing the (003), (006), (012), (018), and (110) peaks of hydrotalcite. The decreased crystallinity was attributed to random stacking of LDH nanolayers and large organic moieties, implying the incorporation of extract inside the LDH nanostructures (Kim et al., 2016).

**FIGURE 3:**
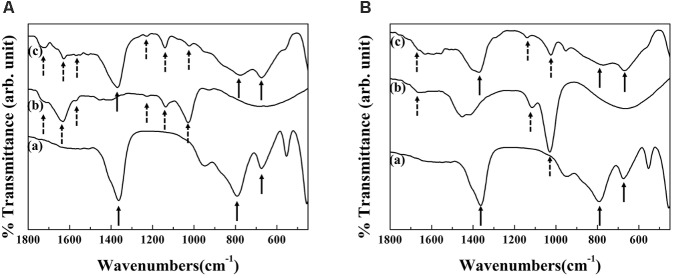
Fourier transform-infrared spectra of (a) MgAl-LDH, (b) extract and (c) hybrid for **(A)** RT-LDH and **(B)** FL-LDH hybrid. LDH, Layered double hydroxide; DS, Decursin species; RT, Root; FL, Flower.

The FT-IR spectrum of RT extract showed the characteristic bands (dashed arrows) of polysaccharide and DS such as α-pyrone ring (1717 cm^−1^), aromatic C=C (1632 and 1566 cm^−1^), C-O stretching (1226 cm^−1^) and aromatic C-O (1136 and 1028 cm^−1^) (**Figure 3A**) (Li et al., 2014; Kim et al., 2016). The characteristic peak of MgAl-LDH (solid arrow) was exhibited at 1365 cm^−1^ (stretching vibration of interlayer CO_3_^2−^) and 790, 674 cm^−1^ (both for M-OH stretching of LDH). After hybridization, all characteristic bands of RT extract and MgAl-LDH were observed at similar peak position with RT and MgAl-LDH. In case of FL extract, -OH bending of polysaccharide and aromatic C-O stretching of DS were exhibited at 1664 cm^−1^ and 1117, 1029 cm^−1^, respectively (**Figure 3B**). IR spectrum of FL-LDH hybrid indicated that the characteristic peak of FL and MgAl-LDH were also preserved after hybridization.

**FIGURE 4:**
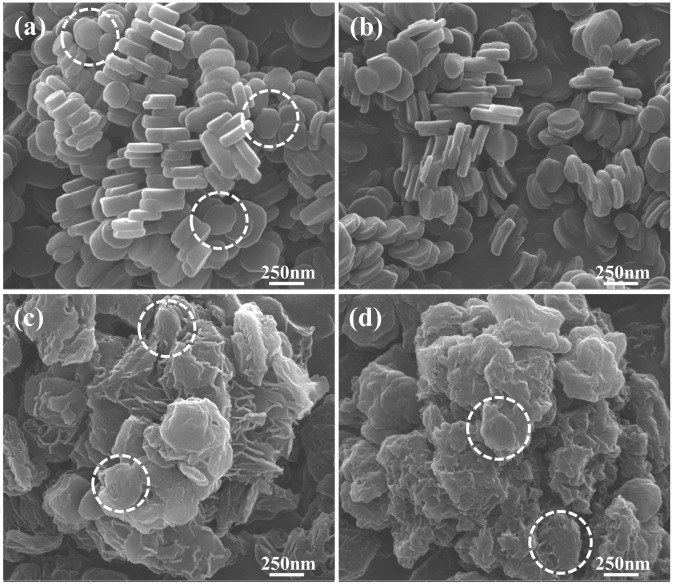
Scanning electron microscopic images of **(a)** MgAl-LDH, **(b)** calcined LDH, **(c)** RT-LDH hybrid, and **(d)** FL-LDH hybrid. Dotted circles stand for primary particles. LDH, layered double hydroxide; RT, root; FL, flower.

Scanning electron microscopic (SEM) images showed step-by-step morphological changes of LDH from pristine to AGN-LDH hybrids. Pristine LDH (**Figure 4a**) exhibited particles having a thick coin-like shape with thickness of ∼90 nm and a diameter of ∼244 nm, which corresponded to the hydrodynamic radius, 255 nm, of MgAl-LDH (Supplementary Figure S1A). Upon calcination, the thickness of the particle was reduced to ∼50 nm, while the diameter and shape were mostly maintained (**Figure 4b**). After hybridization, the overall morphology of particles changed significantly. Both RT-LDH and FL-LDH hybrids demonstrated sand-rose shapes, in which very thin layers were partially bent and agglomerated (**Figures 4c,d**). Although the images for both hybrids seemed to have assembly of particles, close observation exhibited that the primary particle size of pristine LDH, RT-LDH, and FL-LDH hybrid were comparable to each other showing 244, 241, and 239 nm (dashed circles in **Figure 4**), respectively. The hydrodynamic radii of both hybrids in aqueous suspension were 595 and 826 nm for RT-LDH and FL-LDH (Supplementary Figures S1B,C), respectively, suggesting the possibility of agglomerate with two or three particles in hybrids.

**FIGURE 5:**
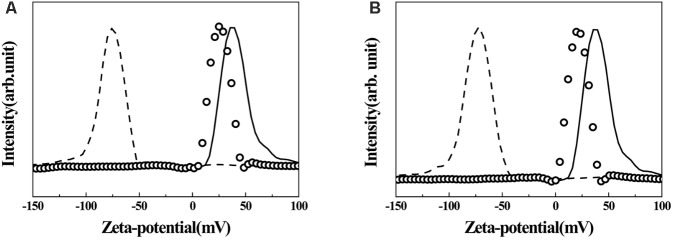
Zeta-potential graphs of AGN-LDH hybrids. **(A)** RT-LDH hybrid and **(B)** FL-LDH hybrid. Solid line: MgAl-LDH, dashed line: AGN extract, open circles: AGN-LDH hybrids. AGN, *Angelica gigas* Nakai; LDH, layered double hydroxide; RT, root; FL, flower.

Surface charges of pristine LDH, AGN extract, and hybrids were investigated by measuring their zeta potentials in an aqueous media of pH ∼7.0. As shown in **Figure 5**, pristine LDH was determined to have a high positive surface charge of ∼+38.5 mV, and the distribution of zeta potential lay in the range of +10 ∼ +75 mV (solid line). Different from pristine LDH, zeta potential values of both AGN extracts (RT and FL) showed strong negative charges centered at ∼−75.6 mV, and the distribution was from −50 to −130 mV. After hybridization, zeta potential value again shifted to the positive region. For both hybrids, the distribution lay between 0 and +50 mV with an average value of +26.7 mV.

**Table 2** displays the DS quantification results of the hybrids. First, the extract content was 18.4 and 16.1 wt/wt% for RT-LDH and FL-LDH hybrids, respectively. The content of DS was 11.5 and 2.23 wt/wt% for RT-LDH and FL-LDH hybrids, respectively. DS content in the original extracts was 30.8 and 6.27 wt/wt% in AGN root and flower, respectively. When we estimated DS content in total extract inside hybrids, they were much larger than those in the original extract, 62.8 wt/wt% and 13.8 wt/wt% for RT-LDH and FL-LDH, respectively.

**Table 2:** Loading capacity and DS content for AGN extract and DS in AGN-LDH hybrids.

	Extract loading capacity in hybrid [extract/hybrid (wt/wt%)]	DS loading capacity in hybrid [DS/hybrid (wt/wt%)]	DS content in total extract [DS/extract (wt/wt%)]
Root extract	–	–	30.8 ± 0.12
Flower extract	–	–	6.27 ± 0.13
RT-LDH	18.4 ± 1.28	11.5 ± 0.05	62.8 ± 4.28
FL-LDH	16.1 ± 0.08	2.23 ± 0.004	13.8 ± 4.73

*AGN, Angelica gigas Nakai; DS, decursin species; LDH, layered double hydroxide; RT, root; FL, flower.*

### Evaluation on DS Release From Hybrids

**FIGURE 6:**
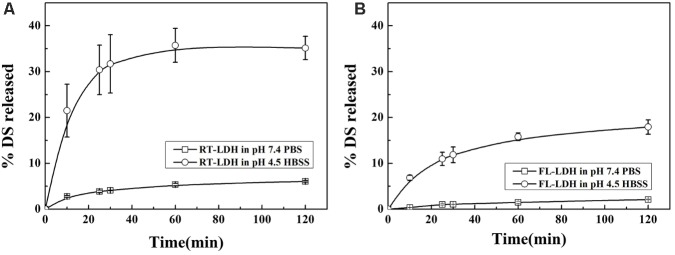
Release patterns of DS from **(A)** RT-LDH hybrid and **(B)** FL-LDH hybrid in pH 7.4 PBS and pH 4.5 HBSS. DS, Decursin species; RT, Root; FL, Flower; LDH, Layered double hydroxide; PBS 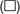, Phosphate buffered saline; HBSS 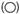, Hanks’ balanced salt solution.

Time-dependent release profiles of DS from hybrids in either neutral physiological condition (pH 7.4 PBS) or lysosomal condition (pH 4.5 HBSS) are displayed in **Figure 6**. RT-LDH slowly released the DS in pH 7.4 PBS, and the cumulative release reached ∼6.1% after 120 min (**Figure 6A**). In contrast to the release profile in pH 7.4 PBS, RT-LDH in pH 4.5 HBSS showed fast release at the early stage (0–25 min), followed by slow and sustained release. The cumulative DS release was almost 35% at 120 min, and the initial burst of DS before 25 min was responsible for ∼30% of the total release (**Figure 6A**). The FL-LDH hybrid showed similar patterns with RT-LDH both in pH 7.4 PBS and pH 4.5 HBSS with more suppressed DS release (**Figure 6B**). The cumulative DS release in the early stage before 25 min was ∼1.0 and 11% in pH 7.4 PBS and pH 4.5 HBSS, and the cumulative release at 120 min was ∼2.1 and 18%, respectively.

The release profiles were fitted to several well-established kinetic models, such as first order, Elovich, parabolic, and power function equations, and their fitting results are summarized in **Table 3**. DS release patterns of RT-LDH and FL-LDH hybrids in pH 7.4 PBS and pH 4.5 HBSS were well-fitted to the Elovich model, showing coefficient of determination *R*^2^ values larger than 0.98 except pH RT-LDH in pH 4.5 HBSS. Although RT-LDH in pH 4.5 HBSS media showed a relatively lower R^2^ value of 0.8451, the Elovich model was determined to be the most reasonable kinetic model among the fitted models in terms of *R*^2^ value.

**Table 3:** Linear regression results of time-dependent DS release pattern from RT-LDH and FL-LDH nanohybrids for kinetic model fitting.

	Kinetic model	Regression equation	*R*^2^
RT-LDH/pH 7.4 PBS	First order	lnQt = −1.834 × 10^−4^ *t* − 0.008964	0.6988
	Elovich equation	Qt = 0.7376 lnt − 0.05392	0.9950
	Parabolic diffusion	Qt = 5.525 × 10^−3^ *t*^1/2^ + 0.007202	0.9338
	Power function	lnQt = 0.3219 lnt − 0.2413	0.9837
FL-LDH/pH 7.4 PBS	First order	lnQt = −7.173 × 10^−5^ *t* − 0.001484	0.8832
	Elovich equation	Qt = 7.842 × 10^−2^ lnt − 0.1416	0.9897
	Parabolic diffusion	Qt = 2.136 × 10^−3^ *t*^1/2^ + 0.001714	0.9678
	Power function	lnQt = 0.6956 lnt − 4.614	0.9193
RT-LDH/pH4.5 HBSS	First order	lnQt = −9.354 × 10^−3^ *t* − 0.5852	0.5831
	Elovich equation	Qt = 3.256 lnt + 6.210	0.8451
	Parabolic diffusion	Qt = 4.314 × 10^−2^ *t*^1/2^ + 0.5687	0.6766
	Power function	lnQt = 0.1992 lnt + 2.154	0.8132
FL-LDH/pH4.5 HBSS	First order	lnQt = −2.105 × 10^−3^ *t* − 0.1221	0.8898
	Elovich equation	Qt = 0.5092 lnt − 0.4070	0.9920
	Parabolic diffusion	Qt = 4.330 × 10^−2^ *t*^1/2^ + 0.1127	0.9368
	Power function	lnQt = 0.3917 lnt − 1.106	0.9645

*DS, decursin species; RT, root; FL, flower; LDH, layered double hydroxide; PBS, Phosphate buffered saline; HBSS, Hanks’ balanced salt solution.*

The Elovich parameters, *a* and *b*, for two AGN-LDH hybrids in PBS and HBSS conditions are summarized in **Table 4**. Parameter *a* is related to the release in the initial phase; a higher *a* value is related to a larger amount of initial release (Mengel, 2000). Parameter *b* stands for the release rate of a loaded moiety (Jalali, 2006). RT-LDH showed an initial burst effect in pH 4.5 HBSS (*a* = 6.21), while its release in the early stage was suppressed in pH 7.4 PBS (*a* = −0.0539). The release rate of RT-LDH was higher in HBSS condition (*b* = 3.26) than in the PBS (*b* = 0.738). For FL-LDH, the parameter *a* was similar to each other in both types of release media, while the release rate of FL-LDH was higher in pH 4.5 HBSS (*b* = 0.509) than in the pH 7.4 PBS (*b* = 0.00784). Compared with RT-LDH, the initial DS release from FL-LDH was fairly suppressed (*a* = 6.21 and −0.407 for RT-LDH and FL-LDH in HBSS media, respectively), and the release rate was very slow (*b* = 0.00784 and 0.509 for PBS and HBSS).

**Table 4:** Parameters obtained from kinetic fitting results of the Elovich equation for the release of DS from AGN-LDH hybrids in pH 7.4 PBS or pH 4.5 HBSS.

	*a*	*b*
RT-LDH in pH 7.4 PBS	−0.0539	0.738
RT-LDH in pH 4.5 HBSS	6.21	3.26
FL-LDH in pH 7.4 PBS	−0.142	0.00784
FL-LDH in pH 4.5 HBSS	−0.407	0.509

*DS, decursin species; AGN, Angelica gigas Nakai; LDH, layered double hydroxide; PBS, Phosphate buffered saline; HBSS, Hanks’ balanced salt solution; RT, root; FL, flower.*

### Anticancer Efficacy of AGN-LDH Hybrids

**FIGURE 7:**
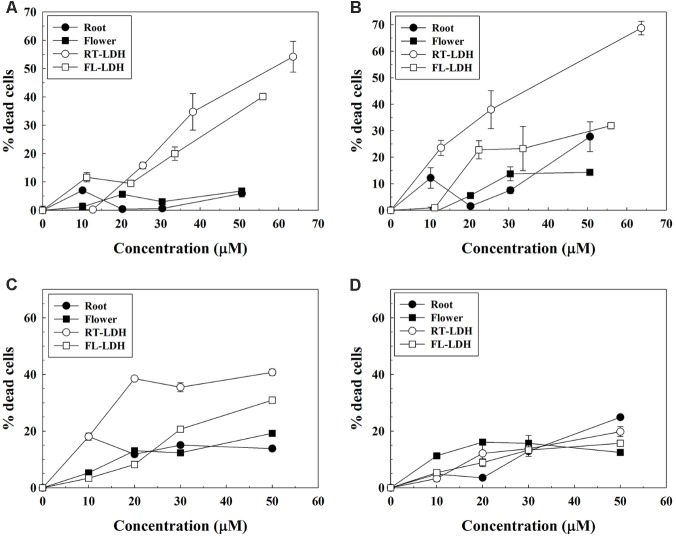
Trypan blue assay of AGN extracts and AGN-LDH hybrids in **(A)** A549, **(B)** HeLa, **(C)** HEK293T, and **(D)** C2C12 cells. *X*-axis means DS concentration. AGN, *Angelica gigas* Nakai; LDH, layered double hydroxide.

Anticancer efficacy of AGN-LDH hybrids was examined in A549, HeLa, and HEK293T cells by trypan blue assay. C2C12 myoblast cells were used as a control. Hybrid sample suspensions were treated based on DS concentration, which was calculated according to the pre-quantified DS content in hybrids. According to the preliminary test, 0.1% DMSO/DMEM solution was found to be non-cytotoxic. As shown in **Figure 7**, AGN extracts from both FL and RT displayed low cancer cell-killing activities. Less than 10% of A549 cells and somewhat more HeLa and HEK293T cells (about 10–20%) were found to be dead at a high DS concentration of 50 μM. However, DS-containing hybrids (FL-LDH and RT-LDH) exhibited much higher cancer cell-killing activities in all cell types than did the AGN extract. Above 20 μM, more cancer cells were found to be dead in the hybrid-treated condition than in the AGN extract only treated condition. Furthermore, RT-LDH showed a higher anticancer activity than FL-LDH in all cell types, with about 40–50% of cancer cells killed at 50 μM in the RT-LDH-treated condition. Interestingly, RT-LDH, and FL-LDH showed low dead cells population (<20%, even at high concentration of 50 mM DS) in C2C12 cells, similarly with root and flower extract (**Figure 7D**).

## Discussion

According to the solubility test (**Table 1**), the organic moieties in the AGN extract including DS can be effectively dissolved utilizing polar aprotic DMSO (**Figure 1**). Amphiphilic DMSO, which has both hydrophobic (two methyl groups) and hydrophilic (sulfoxide group) groups, was expected to effectively dissolve both hydrophilic and hydrophobic chemical species in the extracts. Furthermore, DMSO can facilitate effective reactions between hydrophobic DS and hydrophilic LDH nanomaterials. Thus, DMSO was selected as the hybridization media. The extract/DMSO solutions were determined to have 30.8 and 6.27 wt/wt% of DS for AGN root and flower, respectively, providing contents that were sufficiently high to utilize DS as active components in the anticancer study after hybridizing with LDHs.

Physicochemical analyses such as XRD, FT-IR, SEM, and zeta potential measurement showed that the hybrids possessed AGN extract inside the nanostructures formed by LDH nanoparticles. According to XRD patterns (**Figure 2**), FT-IR spectra (**Figure 3**), and SEM images (**Figure 4**), we conclude that the organic moieties in the AGN extract were incorporated into the LDH nanostructure during the reconstruction process. Crystallinity reduction and peak shift to a lower angle in the hybrid compared with in the pristine LDH indicated that the organic moieties were incorporated. Generally, LDHs undergo partial lattice distortion and re-organization during a reconstruction process (Pérez-Ramírez et al., 2007; Martínez-Ortiz Mde et al., 2008), and this distortion and re-organization could be more significant when large organic molecules, such as those from natural extract, were incorporated (Kim et al., 2016). Diffraction patterns of AGN, both amorphous patterns in the 2𝜃 range 5–30° and several small and sharp peaks, disappeared after hybridization, confirming that the molecular arrangement of the AGN extract was re-organized by host LDH nanostructures. Through FT-IR spectra, organic moieties such as carbohydrate and DS in both extract preserved their intact structure after hybridization. The hydrodynamic radii (Supplementary Figures S1B,C) and primary particle size (**Figures 4c,d**) of both hybrids showed that several LDH particles might be assembled without serious aggregation. A sand-rose morphology consisting of randomly stacked layers of LDH was also found in a previous study when organic moieties of a natural extract were incorporated into LDH nanostructures (Kim et al., 2016). Random stacking of thin LDH layers (∼19.4 nm thickness from **Figure 4**) could form pores able to accommodate the extract. Zeta potential results also supported the conclusion that AGN extract was trapped inside the nanostructures. Zeta potential values of both RT-LDH and FL-LDH were close to those of pristine LDH, showing positive values (**Figure 5**). If the AGN extract covered only the surfaces of LDH nanostructures, the surface charge of the hybrids would be closer to that of AGN extract. Thus, we suggest that LDHs accommodated organic moieties inside the inter-particle pores rather than at its surface. Although LDH can accommodate negatively charged molecules in its interlayer space, we excluded this possibility because the XRD pattern of the hybrid (**Figure 2**) did not show significant interlayer expansion [(003) peak position].

The contents of AGN extract in the hybrid were 18.4 and 16.1 wt/wt% for RT-LDH and FL-LDH, respectively. These contents were larger than those reported in our previous study, where we incorporated AGN root extract into LDH utilizing methanol solvent (Kim et al., 2016). We are fairly sure that the DMSO solvent played an important role in AGN incorporation; it did not only solubilize as much AGN as possible but also guided them to the LDH nanostructure resulting in efficient hybridization. It was worthy to note that the DS content of extract in hybrids was dramatically enhanced compared with the DS content in the extract itself, showing 62.8 and 13.8 wt/wt% in RT-LDH and FL-LDH hybrids, respectively. This result indicates that 62.8% of total organic moiety in the RT-LDH hybrid (13.8% for FL-LDH) was DS, which has anticancer activity. Therefore, we expect that both hybrids would exhibit high anticancer efficacy as nanomedicines.

Both hybrids showed DS release patterns fitted to the Elovich model, which explained adsorption or desorption of adsorbate mediated by bulk surface diffusion. According to the literature, organic molecules encapsulated in LDHs followed the power function model, which hypothesized release mediated by flat surface diffusion or ion exchange (Kang et al., 2015). The Elovich model in this study suggested that the DS moiety was located in the 3-dimensional inter-particle space rather than being arranged in the flat interlayer space. This point is important for anticancer drug release from nanoparticles as the arrangement affects drug release kinetics. When drug molecules are located as a quasi-bulk state in large inter-particle pores as in current sample, a large amount of drug moiety can be easily released to act as anticancer agents. Thus, incorporation of DS moieties in the inter-particle space in the present hybrids and Elovich release could be advantageous to anticancer nanomedicine. It was also noteworthy that the both RT-LDH and FL-LDH hybrids showed enhanced drug release in pH 4.5 HBSS solution, which simulates the lysosomal condition in cancer cells. In this condition, LDH can be partially dissolved into simple ions, thus release of loaded drug is accelerated. As it is known that drug-LDH hybrids are internalized into cells via endocytosis (Oh et al., 2006a), lysosomal escape of DS moiety from both RT-LDH and FL-LDH hybrids is expected with increased DS amount compared with simulated physiological condition (PBS). FL-LDH show suppressed DS release amount in both pH 7.4 PBS and pH 4.5 HBSS compared with RT-LDH, possibly due to the higher organic moieties content in FL-LDH than in RT-LDH (13.8% DS and 86.2% of other organic moieties, respectively, in FL-LDH compared with 62.8% DS and 37.2% in RT-LDH). Major organic components such as lipid, carbohydrate, etc. are thought to trap DS moiety and retard its release. Nevertheless, we conducted anticancer assays for both hybrids, expecting enhanced DS release from the both hybrid in lysosomal condition.

From the literature, DS is known to inhibit cell cycle progression and induce apoptosis in human prostate and murine colon cancer cells (Yim et al., 2005; Son et al., 2011). In addition, decursin and decursinol angelate have been reported to have anti-cancer effects against human cervical cancer cells but not lung cancer cells (Yim et al., 2011). We used human cervical (HeLa), lung (A549), and embryonic kidney cancer cells (HEK293T) for this assay in order to examine the anti-cancer activity of decursin-containing AGN-LDH hybrids on various cell types. Anticancer efficacy test results (**Figure 7**) showing higher percentages of dead cells for treatment with hybrids than for extract only, could be easily explained by a more efficient cellular uptake mechanism and facilitated release of DS in the lysosomal condition. Anti-proliferating activity of AGN-LDH was also examined in C2C12 myoblast cells (**Figure 7D**). It was known that non-transformed “normal” cells were less sensitive to DS. This low cytotoxicity of AGN-LDH in C2C12 cells displays its safety to normal cells. Extracts showed relatively high anti-cancer activity in HeLa cells compared with A549 cells, which is consistent with the results of a previous report (Yim et al., 2011). However, interestingly, LDH hybrids showed significant anticancer activity in all cell lines, depending on the concentration used. Decursin species in extract (closed circles in **Figure 7**) are thought to penetrate the cell membrane by passive diffusion or facilitated transport. However, DS can massively enter cancer cells via LDH-assisted endocytosis and can be released to the cytosol from LDH in acidic lysosomal conditions. In addition, A549 cells result suggests that cytosolic delivery of DS by encapsulation into LDH nanocarriers also may avoid cellular efflux system, contrary to DS only. It should be noted that the amounts of DS in both hybrid and extract were set to be almost the same, and that LDH itself is known to be highly compatible with various cancer cell lines (Choi et al., 2007, 2009). In Supplementary Figure S2, minimal cytotoxicity of LDH itself was also confirmed by MTT assay. Thus, the enhanced anticancer efficacy of hybrids was thought to be attributed to the hybridization of DS with LDHs, as reported previously for drug-LDH hybrids (Oh et al., 2006c; Kim et al., 2013, 2014), and this study verified that the drug-LDH strategy as a nanomedicine can be applied to a natural extract. The fate of LDH, during and after delivering therapeutic agents to cells, is not fully comprehended; however, we can suggest several processes according to the literatures. In our previous work based on electron microscopy (tracing LDH part) and confocal microscopy (tracing encapsulated drug molecules), drug encapsulated LDH enters cell, releases drugs to the cytosol and is excreted out of the cell after 24 h (Oh et al., 2011). The other literature also reported that LDHs could be taken up by cells through endocytosis and finally exocytosed depending on particle size (Chung et al., 2012). The report also claimed that LDH particles could be degraded in lysosome which fused with LDH-containing endosome. To summarize, once entered LDH undergoes partial degradation and exocytosis. Between the two hybrids investigated, RT-LDH showed higher cancer cell suppression effects than did FL-LDH, even though the same quantities of DS were present in both hybrids. This result can be explained by the release behaviors; RT-LDH exhibited ∼35% release within 120 min, while FL-LDH released ∼18% in this same amount of time. Thus, facilitated DS release from RT-LDH in the lysosomal condition is thought to induce effective anticancer activity.

## Conclusion

We prepared hybrids between LDH and AGN root or flower extract containing DS, anticancer phytochemicals, via the reconstruction route. In order to effectively solubilize DS and to facilitate hybridization with LDH, polar aprotic DMSO solvent was used. According to the XRD patterns, FT-IR spectra, SEM images, and zeta potential values, the AGN-LDH hybrid possessed AGN extract inside the nanostructured pores developed by random stacking of LDH nanoparticles. Quantitative analyses revealed that 15–20 wt% of extract was incorporated into AGN-LDH hybrids, and DS in AGN-LDH hybrid was twice as concentrated as that in AGN extract itself. The release profiles of both AGN-LDH in pH 7.4 PBS and pH 4.5 HBSS fitted well with the Elovich equation, indicating bulk surface release, suggesting that the DS located in the inter-particle pore of LDH was released in a bulk surface manner. Cell viability tests by trypan blue assay in cancer cells demonstrated the dramatically enhanced anticancer activity of the AGN-LDH hybrid compared with AGN itself. Between the two hybrids examined, RT-LDH was determined to have higher anticancer activity than FL-LDH, and this difference was attributed to the facilitated DS release from RT-LDH in the lysosomal condition.

## Author Contributions

H-JK carried out the preparation, characterization, and release study on AGN-LDH hybrids. GJL performed the anticancer activity tests and interpreted data. A-JC prepared AGN extract and evaluated the solubility and bio-active components of AGN extract. T-HK analyzed characterization data of AGN-LDH hybrids. T-iK designed the anticancer activity evaluation and drafted the manuscript. J-MO designed the material preparation and also drafted the manuscript. All authors read and approved the final manuscript.

## Conflict of Interest Statement

The authors declare that the research was conducted in the absence of any commercial or financial relationships that could be construed as a potential conflict of interest.
