# Supplementary material for: Layered Double Hydroxide Nanomaterials Encapsulating Angelica gigas Nakai Extract for Potential Anticancer Nanomedicine
Source: Front Pharmacol. 2018 Jul 9;9:723. doi: 10.3389/fphar.2018.00723 (PMC6046438; doi:10.3389/fphar.2018.00723)
Supplement: Supplementary file 1 [file Data_Sheet_1.DOC]

**Supplementary Material**

# Layered Double Hydroxide Nanomaterials Encapsulating *Angelica Gigas* Nakai Extract for Potential Anticancer Nanomedicine

Fig S1. Hydrodynamic radii of (a) MgAl-LDH, (b) RT-LDH and (c) FL-LDH hybrid in deionized water. In order to measure hydrodynamic radii of MgAl-LDH, RT-LDH and FL-LDH, 0.5 mg of each sample powder was dispersed in deionized water, and then hydrodynamic radii of each sample was evaluated with ELSZ-1000 (Otsuka, Kyoto, Japan).


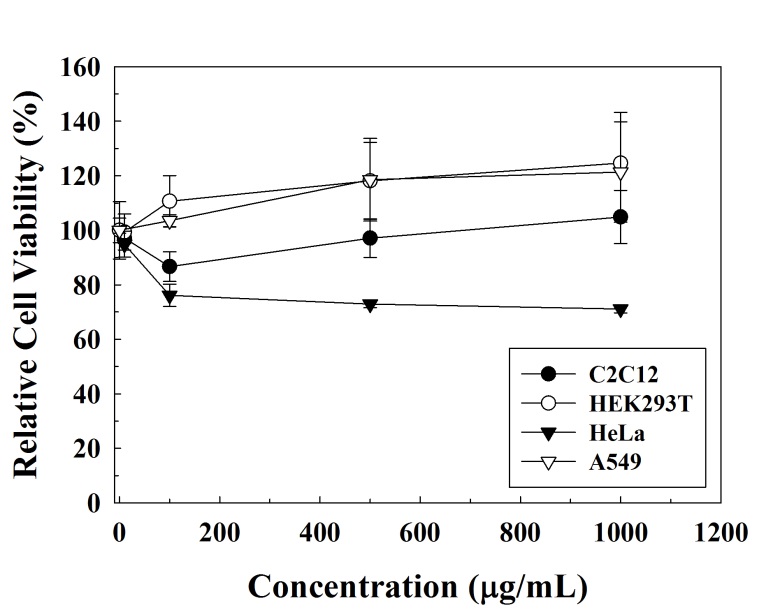
Fig. S2. MTT assay result of LDH only. Cytotoxicity of LDH itself was measured by MTT assay in A549, HeLa, HEK293T, and C2C12 cells. Cells were seeded in 96-well cell culture plates at a density of 1 × 10^4^ cells/well. When achieving 70−80% of confluency after 24 h, the cells were exposed to 100 μL of LDH solutions with various concentrations in serum-containing medium for 24 h. After an exchange of medium with fresh medium containing 10% FBS, the cells were treated with MTT solution (2 mg/mL in DPBS) for 2 h at 37 °C. After removing each medium carefully, the formazan crystal formed by proliferating cell was dissolved in 150 μL of DMSO. The absorbance was measured at 570 nm using a microplate reader. Results were presented as relative cell viabilities (RCV, percentage values relative to value of untreated control cells). All experiments were performed in quadruplicate.
